# Increased abundance of *Firmicutes* and depletion of *Bacteroidota* predicts poor outcome in chronic lymphocytic leukemia

**DOI:** 10.3892/ol.2024.14685

**Published:** 2024-09-17

**Authors:** Magdalena Paziewska, Monika Szelest, Michał Kiełbus, Marta Masternak, Joanna Zaleska, Ewa Wawrzyniak, Aleksandra Kotkowska, Monika Siemieniuk-Ryś, Marta Morawska, Elżbieta Kalicińska, Paula Jabłonowska, Tomasz Wróbel, Anna Wolska-Washer, Jerzy Zdzisław Błoński, Tadeusz Robak, Lars Bullinger, Krzysztof Giannopoulos

**Affiliations:** 1 Department of Experimental Hematooncology, Medical University of Lublin, 20-093 Lublin, Poland; 2 Department of Hematology and Bone Marrow Transplantation, St John's Cancer Centre, 20-090 Lublin, Poland; 3 Department of Hematology, Medical University of Lodz, 93-510 Lodz, Poland; 4 Department of Hematology, Blood Neoplasms and Bone Marrow Transplantation, Wroclaw Medical University, 50-367 Wroclaw, Poland; 5 Department of Experimental Hematology, Medical University of Lodz, 93-510 Lodz, Poland; 6 Department of Hematooncology, Copernicus Memorial Hospital, 93-513 Lodz, Poland; 7 Department of General Hematology, Copernicus Memorial Hospital, 93-513 Lodz, Poland; 8 Department of Hematology, Oncology and Cancer Immunology, Charité-Universitätsmedizin Berlin (Corporate Member of Free University of Berlin, Humboldt University of Berlin), D-13353 Berlin, Germany

**Keywords:** chronic lymphocytic leukemia, microbiome, oral microbiome, gut microbiome, 16S rRNA next-generation sequencing

## Abstract

Evidence indicates that there are significant alterations in gut microbiota diversity and composition in patients with hematological malignancies. The present study investigated the oral and intestinal microbiome in patients with chronic lymphocytic leukemia (CLL) (n=81) and age-matched healthy volunteers (HVs; n=21) using 16S ribosomal RNA next-generation sequencing. Changes in both oral and gut microbiome structures were identified, with a high abundance of *Proteobacteria* and depletion of *Bacteroidetes* in CLL as compared to HVs. Oral and stool samples of patients with CLL revealed a significant change in the abundance of short-chain fatty acid-producing genera in comparison with HVs. Furthermore, the relative abundance of oral and intestine *Bacteroidetes* was significantly decreased in patients with CLL with negative prognostic features, including unmutated immunoglobulin heavy chain gene (IGHV). Notably, an increased abundance of gut *Firmicutes* was found to be associated with high expression of CD38. Finally, the present study suggested the log *Firmicutes/Bacteroidota* ratio as a novel intestinal microbiome signature associated with a shorter time to first treatment in individuals with CLL. The findings indicate that oral and gut microbial diversity in CLL might point to the inflammatory-related modulation of the clinical course of the disease.

## Introduction

Chronic lymphocytic leukemia (CLL) is well-characterized biologically lymphoid malignancy with a remarkably heterogeneous clinical course, which is reflected in varied survival times, response to treatment, and the dynamics of disease progression. To date, much evidence has emerged reporting several novel alterations that elucidate genomic, epigenetic, immunogenetic, and tumor microenvironmental mechanisms, which might drive the evolution of the disease (1–6). Although these advances have vastly expanded the knowledge of CLL pathogenesis, the link between the considerable molecular heterogeneity of this malignancy and the clinical outcome of patients remains elusive.

For the last years it has been speculated that immunologic and inflammatory factors, including antigen stimulation, could be involved in the processes determining the development and progression of CLL (7). The prevalence of CLL increases noticeably with age, implying that a persistent exposure to a self and foreign antigen might be considered as a predisposing factor. Since CLL patients present progressive immunodeficiency, recurrent infections are a common clinical feature of this disease (8). Early studies suggested that gut microbiota plays a key role in defining the B cell receptor (BCR) repertoire (9), thus stimulation of alloantigens derived from distinct microbial species might be involved in the development and proliferation of CLL-specific B cell clones, and thereby might potentially stand behind the interindividual variability of clinical outcomes. Furthermore, certain types of bacteria release factors that might indirectly contribute to neoplasia by maintaining a proinflammatory environment (10).

The crosstalk between the microbiome and immune system takes place at numerous sites, including the skin and mucosal surfaces. Commensal species within the gastrointestinal mucosa have been shown to contribute to innate as well as adaptive immunity at multiple levels (11). Our previous report documented an accumulation of CD5+CD19+ cells in tonsillar tissue during chronic antigenic stimulation accompanying chronic or recurrent tonsillitis in children (12). Recently, low intestinal microbial diversity and increased abundance of specific bacterial community members have both been reported to be implicated in the induction of gene mutations and host immune response (13).

In the last years, gut microbiota has been proven to modulate the activity of the immune system by creating an imbalance between cell proliferation and apoptosis (14). To date, significant alterations in microbiota diversity and composition have been documented in many cancers, including hematological malignancies, such as acute lymphoblastic leukemia (ALL) (15,16), acute myeloid leukemia (AML) (17,18), and CLL (19). Notably, compelling evidence shows that host microbiota not only influence cellular homeostasis or tumor susceptibility, but also is implicated in disease prognosis and modulation of the efficacy and toxicity of different anti-tumor therapeutic approaches, including chemotherapy, radiotherapy, and immunotherapy (20–23). Therefore, the effects of structural imbalance of host microbiota might contribute to the interindividual variability in treatment response as for immunocompromised patients.

Since the activation of cellular proinflammatory signaling pathways driven by somatic mutations and an increased release of proinflammatory cytokines is associated with CLL development, it seems relevant to investigate the role of chronic inflammation in the pathogenesis of this disease. Notably, analyzing both oral cavity and gut microbiota community structure in CLL patients will allow identifying the microbiota profile related to this proinflammatory environment and define CLL-specific bacterial strains that might recognize microbial patterns that distinguish those patients who do not require treatment and could serve as biomarkers for predicting disease progression and treatment initiation.

## Materials and methods

### Study design and participants

The study was approved by the local Ethics Committee of the Medical University of Lublin (KE-0254/7/2019), and written informed consent forms were obtained from all participants, including CLL patients and healthy volunteers (HVs). The study was performed in accordance with the principles of the Declaration of Helsinki.

Throat swabs, stool, and peripheral blood samples from 81 newly diagnosed and untreated CLL patients were collected. The clinical characteristics of CLL individuals are presented in Table I. As controls, oral and fecal samples from 21 HVs [12 females and 9 males at a median age of 57 years (range 50–84)] were used. The exclusion criteria included antibiotics therapy within four weeks and a history of diarrhea and/or vomiting within 72 h. For HVs, exclusion criteria also included cancer, diabetes, gastrointestinal diseases, and other conditions that could be affecting the microbiome. There were no significant differences in body mass index (BMI) value between CLL patients and HVs (median 27.34, range 20.02–48.46 vs. median 27.30, range 20.07–32.00, P=0.39).

### Peripheral blood mononuclear cells isolation

Peripheral blood mononuclear cells (PBMCs) were isolated from whole blood using density gradient centrifugation on Biocoll (Biochrom, Germany). They were then cryopreserved in RPMI 1640 medium (Biochrom, Germany) supplemented with 20% fetal bovine serum (Biochrom, Germany) and 10% dimethyl sulfoxide (Sigma Aldrich, Germany) and stored at −80°C until further analyses were performed.

### CD38 and ZAP-70 expression analysis

The expression of CD38 and ZAP-70 on CLL cells was assessed by flow cytometry after incubation with monoclonal mouse antihuman antibodies: anti-CD5 PE-Cy5, anti-CD19 FITC, anti-CD38 PE, and anti-ZAP-70 PE (all BD Biosciences, San Jose, CA, USA). Cells were analyzed by FACSuite (BD Biosciences, San Jose, CA, USA) on BD FACS Lyric (BD Biosciences, San Jose, CA, USA). Results were compared to negative control cells without antibodies, and FMO (fluorescence minus one) control in the absence of anti-CD38 PE/anti-ZAP-70 PE monoclonal antibodies. Cut-off points to define CD38+ CLL and ZAP-70+ CLL patients' populations were 30 and 20%, respectively. The gating strategy and representative CD38-positive and ZAP-70-positive samples have been presented in Fig. S1.

### DNA isolation

The QIAamp DNA Bood Mini Kit (Qiagen, Hilden, Germany) for DNA isolation from PBMCs was used according to the manufacturer's instructions. DNA quality and quantity were determined through 260/280 nm absorbance measures using the BioSpec-Nano spectrophotometer (Shimadzu, Kyoto, Japan).

### IGHV and TP53 mutation status assessment

The *TP53* mutation status was determined by PCR amplification of exons 4 to 10 followed by bidirectional Sanger sequencing. The obtained sequences were analyzed using GLASS software (24) according to the ERIC guidelines (25). For IGHV somatic hypermutation status determination, the *IGHV–IGHD-IGHJ* gene rearrangement was amplified using framework region (FR1) primers following BIOMED-2 protocol (26). Then, heteroduplex analysis and bidirectional Sanger sequencing were performed. IMGT/V-Quest software (27,28) was used to analyze the obtained sequence following ERIC guidelines (29). A 98% germline homology cut-off was used to determine IGHV mutational status. The sequences with a germline homology of 98% or higher were considered unmutated, and those with a homology <98% were considered mutated. A subset analysis was performed using ARRest/AssignSubsets software (30).

### Cytogenetic aberrations

Cytogenetic aberrations (del17p, del11q, del13q, tri12, del6q) were assessed by fluorescence in situ hybridization (FISH) in the diagnostic laboratory according to the routine procedures.

### Oral and fecal sample collection, storage, and preparation for microbiome profiling

Throat swabs were collected and stabilized using OMNIgene•ORAL kit (DNA Genotek Inc, Canada). The OMNIgene•GUT kit (DNA Genotek Inc, Canada) was used to self-collect fecal samples by study participants. Both oral and stool samples were stored until shipment to the laboratory according to the manufacturer's recommendations. DNA extraction, amplicon libraries preparation, and 16S rRNA gene sequencing were performed by Eurofins Genomics Europe Sequencing GmbH (Ebersberg, Germany). For target-specific PCR amplification of V3-V5 hypervariable regions of the 16S rRNA gene, the primers V3F (5′-CCTACGGGNGGCWGCAG-3′) and V5R (5′-CCGYCAATTYMTTTRAGTTT-3′) were used. Amplicon libraries covering the specified regions were sequenced on the high-throughput Illumina MiSeq platform (Illumina).

### Microbiome profiling and statistics

Following the quality check with the use of fastqc (31), the dataset was normalized by a subsampling-based strategy using seqtk (32). Reads across all samples were randomly down-sampled to the lowest read count in the cohort. Low-quality ends of the reads were trimmed and filtered using a value of 3 for the maximal error rate parameter. Next, the paired reads were merged. The taxonomic classification for microbiome analysis was determined using the SILVA reference database version 138.1 (33). All the above steps (including reads trimming, filtering, merging, and taxonomic assignment) were performed using the dada2 R package (34). The microbial phylogenetic tree was reconstructed from the obtained sequences using IQ-TREE maximum likelihood phylogeny stochastic algorithm (35).

For data exploration and visualization, including alpha and beta diversity metrics, the set of R packages: phyloseq (36), microbiome (37), microbiomeutilities (38), microbial (39), and microViz (40) was used. Faith's Phylogenetic Diversity (PD) was estimated using picante (41). For comparing microbial communities divided into different sample groups, the Unifrac algorithm (42) and the Bray-Curtis dissimilarity approach (43) were used, for which also non-metric multidimensional scaling (NMDS) plots were generated. Differences in beta diversity were assessed using Permutational multivariate analysis of variance (PERMANOVA) implemented into the vegan R package (44). Linear discriminant analysis (LDA) was taken advantage of to compare the relative abundance of the different taxa between sub-groups (45). The log_FB metric was defined for each sample as the log of *Firmicutes* to *Bacteroidota* relative abundance ratio. The survival package (46) was used to perform the Cox Proportional Hazards Regression models for the assessment of the HR and 95% CI to test the association of selected factors with time to first treatment (TTFT). Cutpoints for continuous variable metrics were evaluated by maximally selected rank statistics with the use of the maxstat R package (47) implemented by the survminer R tool (48). Testing group differences included a two-tailed Wilcoxon test. Survival probabilities were estimated with the use of Kaplan-Meier method and compared using the long-rank test. P-value <0.05 was considered statistically significant. Statistical analyses were performed using R software version 4.1.3 (49).

**Figure 1.**
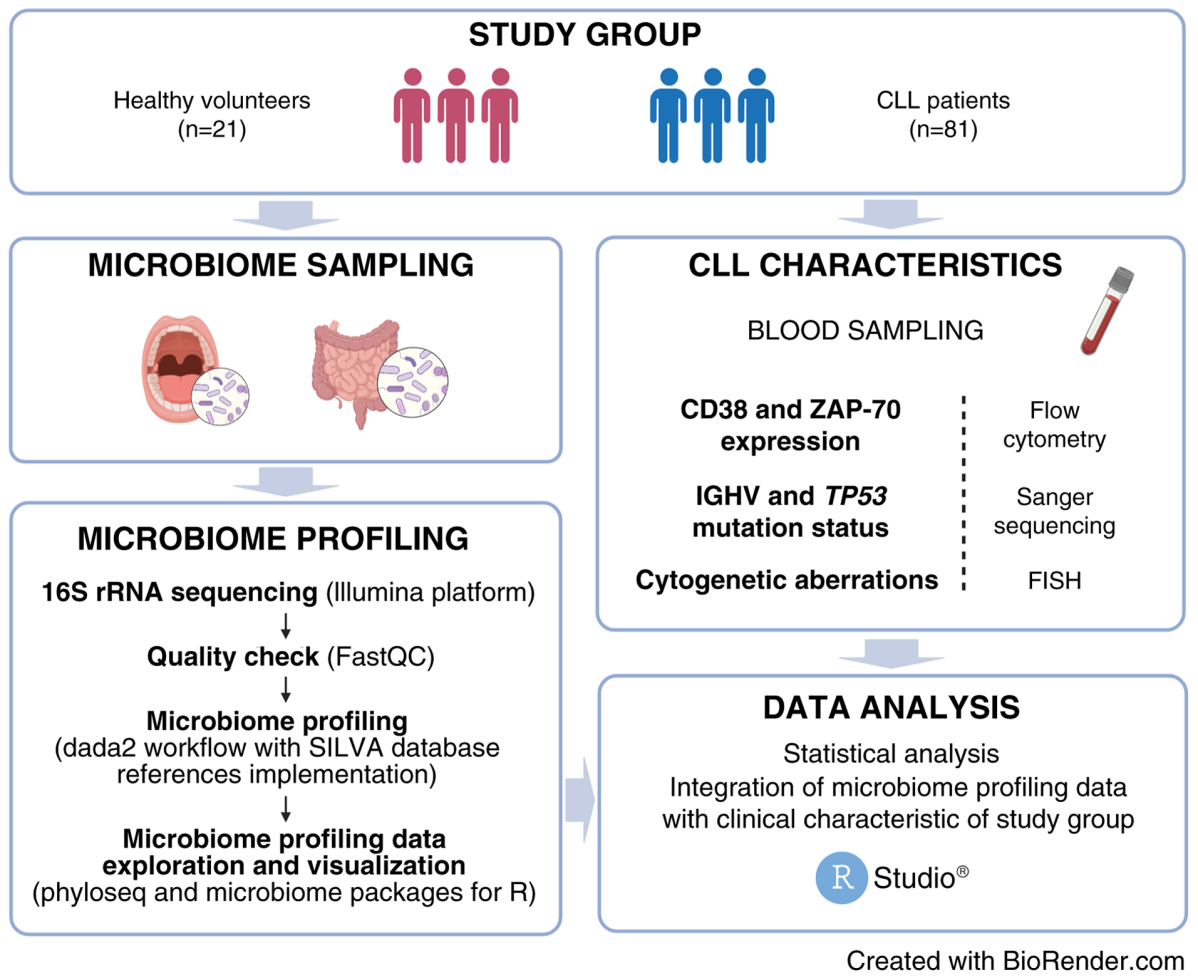
Workflow for oral and gut microbiome analysis in patients with CLL and HVs in the present study. Figure created with BioRender.com. CLL, chronic lymphocytic leukemia; HVs, healthy volunteers.

A flowchart illustrating workflow for oral and gut microbiome analysis in CLL patients and HVs in our study has been presented in Fig. 1.

## Results

### Microbiota structure in patients with CLL and HVs

The microbiota composition of 69 oral and 75 fecal samples from 81 CLL patients and 17 oral and 21 stool samples from 21 HVs were all analyzed. The optimized sequences were obtained through data quality control and read preprocessing, and a total of 17.8 k operational taxonomic units (OTUs) were annotated. Among these identified OTUs, unique annotations were used for further analysis and classified into 23 phyla, 46 classes, 103 orders, 211 families, 585 genera, and 957 species.

### Alpha-diversity and beta-diversity analysis

**Figure 2.**
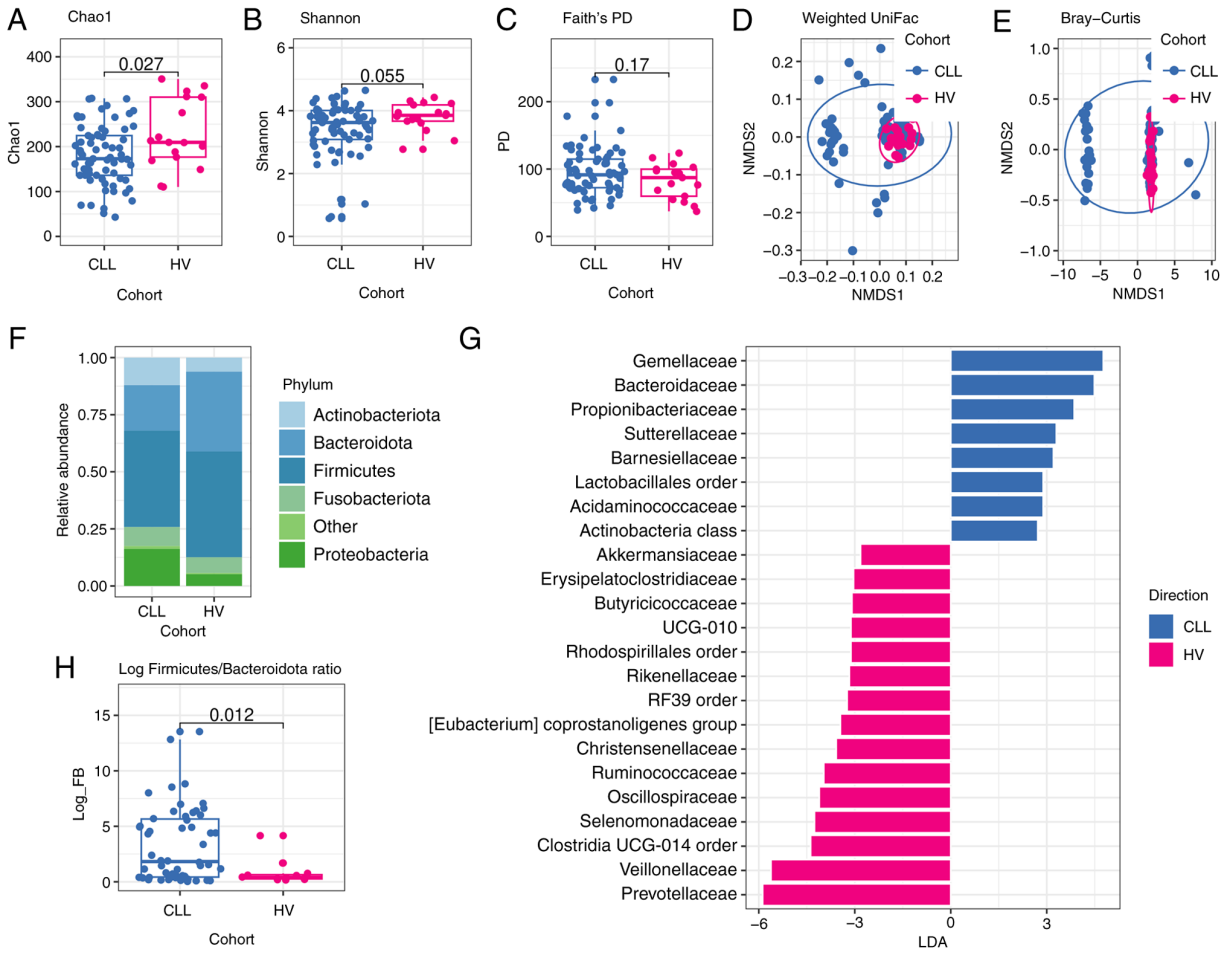
Comparison of oral microbiota of patients with CLL and HVs. (A) Microbial richness index of Chao1; (B) Microbial diversity index of Shannon; (C) Microbial diversity index of Faith's PD. P-values shown in Fig. A-C were calculated by a two-sided Wilcoxon rank-sum test without adjustment of multiple comparisons for CLL (n=69) vs. HVs (n=17). NMDS analysis based on (D) weighted UniFrac distance (R^2^=0.093; P=0.002) and (E) Bray-Curtis dissimilarity (R^2^=0.081; P=0.002). P-values corresponding to D and E figures were analyzed using the PERMANOVA test (as implemented by the vegan R package), whereas dots represent samples. (F) Phylogenetic composition of oral samples at the phylum level; phyla with a relative abundance <0.1% in each sample are merged into ‘Other’; (G) LEfSe analysis indicates enriched bacterial families associated either with CLL (blue; n=69) or HVs (magenta; n=17). The length of the bar column represents the LDA score. The logarithmic LDA scores threshold was 2.0, P<0.05 (a two-sided Wilcoxon rank-sum test without adjustment of multiple comparisons was used for P-value calculation). (H) Log *Firmicutes/Bacteroidota* (log FB) ratio. The P-value was calculated by a two-sided Wilcoxon rank-sum test without adjustment of multiple comparisons for CLL (n=69) vs. HVs (n=17). CLL, chronic lymphocytic leukemia; HVs, healthy volunteers; PD, phylogenetic diversity; NMDS, non-metric multidimensional scaling; LDA, Linear discriminant analysis; LEfSe, LDA effect size.

The microbiota diversity within a single sample is reflected by alpha-diversity, specifically Chao1 and Shannon indexes. These non-phylogenetic metrics revealed that CLL oral samples are characterized by a lower richness and evenness than matched control (Chao1 index median 173.0 vs. median 209.5, P=0.027; Shannon index median 3.62 vs. median 3.85, P=0.055). According to Faith's phylogenetic diversity (PD) metric, which is based on the phylogenetic relationships of microbial taxa, there were no significant differences in the diversity of oral microbiome between CLL patients and HVs (median 91.41 vs. median 87.28, P=0.17), (Fig. 2A-C).

**Figure 3.**
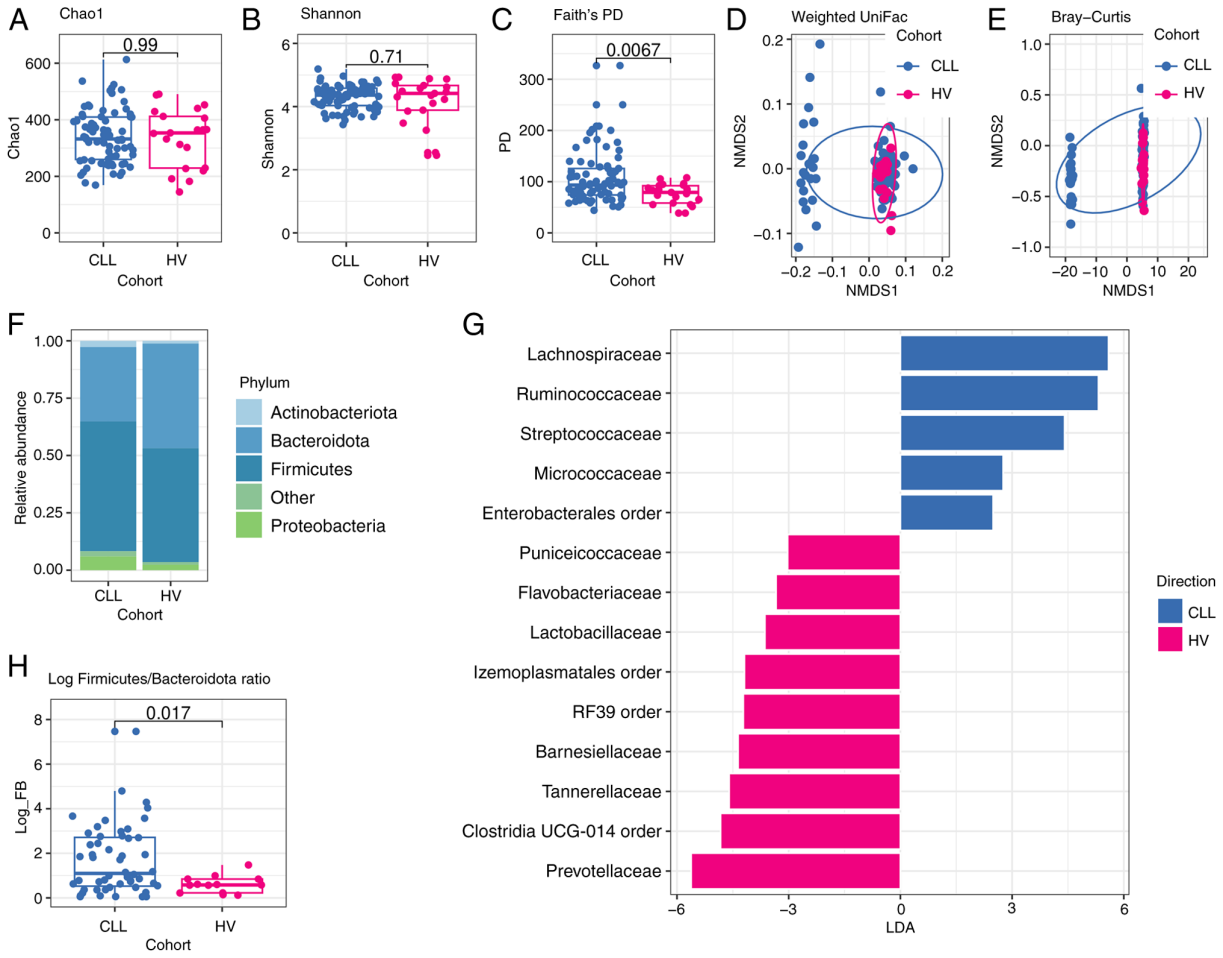
Comparison of gut microbiota of patients with CLL and HVs. (A) Microbial richness index of Chao1; (B) Microbial diversity index of Shannon; (C) Microbial diversity index of Faith's PD. P-values shown in Fig. A-C were calculated by a two-sided Wilcoxon rank-sum test without adjustment of multiple comparisons for CLL (n=75) vs. HVs (n=21). NMDS analysis based on (D) weighted UniFrac distance (R^2^=0.038; P=0.023) and (E) Bray-Curtis dissimilarity (R^2^=0.037; P=0.009). P-values corresponding to D and E figures were analyzed using the PERMANOVA test (as implemented by the vegan R package), whereas dots represent samples. (F) Phylogenetic composition of stool samples at the phylum level; phyla with a relative abundance <0.1% in each sample are merged into ‘Other’; (G) LEfSe analysis indicates enriched bacterial families associated either with CLL (blue, n=75) or HVs (magenta, n=21). The length of the bar column represents the LDA score. The logarithmic LDA scores threshold was 2.0, P<0.05 (a two-sided Wilcoxon rank-sum test without adjustment of multiple comparisons was used for P-value calculation). (H) Log *Firmicutes/Bacteroidota* (log FB) ratio. The P-value was calculated by a two-sided Wilcoxon rank-sum test without adjustment of multiple comparisons for CLL (n=75) vs. HVs (n=21). CLL, chronic lymphocytic leukemia; HVs, healthy volunteers; PD, phylogenetic diversity; NMDS, non-metric multidimensional scaling; LDA, Linear discriminant analysis; LEfSe, LDA effect size.

Furthermore, no significant differences in species richness and evenness between CLL and HVs stool samples (Chao1 index median 332.06 vs. median 353.50 P=0.99, Shannon index median 4.36 vs. median 4.42 P=0.71) were found. However, Faith's phylogenetic diversity (PD) metric revealed that the gut microbial community of CLL patients is more evolutionarily distinct in comparison to HVs (median 93.42 vs. median 79.09 P=0.0067) (Fig. 3A-C).

Next, NMDS was performed for beta-diversity analysis of oral and gut microbial community structure. The result indicated that the structure of the oral microbiome in CLL patients was significantly different from that of HVs group based on Bray-Curtis dissimilarity (R^2^=0.081, P=0.002) and on unweighted uniFrac distance (R^2^=0.06, P=0.003). Moreover, the differences were significant based on weighted uniFrac distance (R^2^=0.093, P=0.002) (Fig. 2D, E). For beta-diversity of the gut microbiome as determined by Bray-Curtis dissimilarity and UniFrac distances, significant differences between CLL and HVs were found, P=0.009 for Bray-Curtis (R^2^=0.037), P=0.001 for unweighted uniFrac (R^2^=0.061), and P=0.023 for weighted uniFrac (R^2^=0.038) (Fig. 3D and E).

### A significant change in the composition and abundance of oral and gut microbiome in CLL patients

The representative sequences of OTUs were compared with the SILVA microbial reference database as to obtain information on the species classification corresponding to each OTU. CLL patients differ from HVs in the observed community structure. The predominant phylum among CLL oral microbiome was *Firmicutes* (42.25%), followed by *Bacteroidota* (19.89%), *Proteobacteria* (16.22%), *Actinobacteriota* (12.05%), and *Fusobacteriota* (8.35%) (Figs. 2F, S2A), while almost 90% of the CLL fecal-derived bacteria were classified into two dominant phyla: *Firmicutes* (56.62%) and *Bacteroidota* (32.43%), followed by *Proteobacteria* (6.01%) and *Actinobacteria* (2.71%) (Figs. 3F, S2C).

### The structure of the oral microbiota in CLL patients and HVs

Interestingly, remarkable differences in the relative abundances of specific bacterial phyla in both oral and intestinal microbiome between CLL patients and HVs were observed. The *Proteobacteria*, a common feature of dysbiosis, was significantly more abundant in CLL oral samples in comparison to HVs oral samples (P=0.022), whereas the abundance of *Bacteroidota* was significantly lower in CLL oral samples compared to HVs oral samples (P=0.0015) (Table SI). Furthermore, a significant difference in the value of log *Firmicutes* and *Bacteroidota* (log F/B) ratio was found between CLL patients and HVs (0.81 vs. 0.28, P=0.012) (Fig. 2H).

Linear discriminant analysis (LDA) effect size (LEfSe) analysis revealed significant bacterial differences in oral microbiota between the CLL patients and HVs. In particular, at the family level, a significantly higher abundance of *Gemellaceae, Bacteroidaceae, Propionibacteriaceae*, and *Sutterellaceae*, as well as depletion of *Prevotellaceae, Veillonellaceae, Oscillospiraceae*, and *Ruminococcaceae*, were observed among oral CLL samples in comparison to HVs (Fig. 2G). As *Prevotellaceae, Veillonellaceae*, and *Ruminococcaceae* have been reported to produce short-chain fatty acids (SCFA) involved in immunomodulation, the differences in the relative abundance of genera belonging to these taxa in the oral microbiome between CLL and HVs were analyzed. CLL oral samples demonstrated a significantly lower abundance of *Prevotella* and *Veilonella* genera (P=0.0011 and P=0.0016, respectively) in comparison to HVs. Additionally, a tendency to higher relative abundance of *Rothia (Micrococcaceae* family) and *Fusobacteria (Fusobacteriaceae* family) in CLL oral samples in comparison to HVs oral samples (P=0.067 and P=0.097, respectively) was ascertained.

### The structure of the gut microbiota in CLL patients and HVs

Similarly to oral samples, fecal samples from CLL patients exhibited an increased abundance of *Proteobacteria* and a decreased abundance of *Bacteroidota* in comparison to fecal samples collected from HVs (P=0.045 and P=0.026, respectively) (Table SII). Consequently, the value of the log F/B ratio was significantly higher in fecal samples from CLL compared to HVs (0.62 vs. 0.22, P=0.017) (Fig. 3H).

LEfSe analysis of gut microbiota indicated significant differences in the abundance of SCFA producers between CLL patients and HVs. CLL fecal samples exhibited an enrichment of *Lachnospiraceae, Ruminococcaceae*, and *Streptococcaceae* families, while HVs fecal samples were enriched in *Prevotellaceae, Tannerellaceae* and *Barnesiellaceae* families (Fig. 3G). At the genus level, CLL fecal samples showed a significantly higher abundance of *Roseburia* (*Lachnospiraceae* family) in comparison to HVs (P=0.011).

### A significant change in the composition and abundance of oral and gut microbiome in CLL patients with respect to the selected prognostic and predictive features

Of note, specific alterations in the oral and intestinal microbiome of CLL patients with different status of selected prognostic features, such as Binet stage, mutation status of *TP53* and IGHV, the presence of cytogenetic aberrations, and expression levels of CD38 and ZAP-70, were found (Tables SIII and SIV).

### Microbial diversity in oral microbiota in CLL patients with respect to the selected prognostic and predictive features

Oral samples from CLL patients with Binet stage A showed a lower relative abundance of *Actinobacteriota* and *Fusobacteriota* and a tendency to present a higher relative abundance of *Bacteroidetes* compared to CLL patients with Binet stage B (P=0.041, P=0.047, P=0.06 respectively). At the family level, oral samples from CLL patients with Binet stage A were more abundant in *Prevotellaceae* compared to CLL patients with Binet stage B (P=0.022) and C (P=0.07), and less abundant in *Lachnospiraceae* and *Fusobacteriaceae* in comparison to oral samples from CLL patients with Binet stage B (P=0.022, P=0.035). Moreover, *Actinobacteriota* showed a tendency to a higher abundance in oral microbiota in CD38+ CLL patients in comparison to CD38-CLL patients (P=0.061). CLL patients with unmutated IGHV showed a tendency to the decreased relative abundance of *Bacteroidetes* phylum and *Prevotellaceae (Bacteroidetes* phylum), as well as *Veillonellaceae* (*Firmicutes* phylum) families in oral samples compared to CLL patients with mutated IGHV (P=0.077, P=0.087, and P=0.053, respectively). Interestingly, CLL patients with stereotyped subsets exhibited enrichment in *Proteobacteria* in comparison to non-stereotyped CLL patients (P=0.016).

However, there were no significant alterations in oral microbiome composition of CLL patients with distinct *TP53* mutation status or the presence of del13q and del17p. Nevertheless, an increased relative abundance of *Proteobacteria* and a tendency to the higher relative abundance of *Fusobacteriota* was found in oral samples from CLL patients with del11q compared to samples from patients with no del11q (P=0.019 and P=0.071, respectively). Moreover, CLL patients with tri12 exhibited an increased abundance of *Actinobacteriota* and *Firmicutes* (P=0.048 and P=0.028, respectively) phyla and a tendency to lower abundance of *Fusobacteriaceae* family (P=0.088) as related to patients with no tri12.

### Microbial diversity in gut microbiota in CLL patients with respect to the selected prognostic and predictive features

Stool samples from patients with Binet stage A exhibited an increased abundance of *Bacteroidetes* and a decreased abundance of *Firmicutes* in comparison to stool samples from patients with Binet stage B (P=0.0069 and P=0.047, respectively) and C (P=0.038 and P=0.067, respectively). At the family level, *Bacteroidaceae* was more abundant in stool samples from patients with Binet stage A compared to Binet stage B (P=0.051) and C (P=0.022), whereas *Prevotellaceae* was less abundant in stool samples from patients with Binet stage A compared to Binet stage B (P=0.02).

Notably, the gut microbiome of CD38+ CLL patients exhibited a significant increase of *Firmicutes* phylum (P=0.045) and a decrease in the *Bacteroidaceae* family (P=0.045) in comparison to CD38-CLL. Moreover, a tendency to the decreased relative abundance of *Bacteroidetes* phylum and *Bacteroidetes* family was found in fecal samples from CLL patients with unmutated IGHV compared to mutated IGHV (P=0.079, P=0.089, respectively). There were no significant differences in gut microbiome composition of CLL patients with distinct *TP53* mutation status or the presence of del17p, del11q, and tri12.

### Log F/B ratio of the gut microbiota as a potential prognostic feature

Notably, there was a significant increase in log F/B ratio in CLL patients with Binet stage B and Binet stage C compared to Binet stage A (P=0.012 and P=0.038, respectively). Furthermore, a tendency to a higher log F/B ratio was found in CLL patients with unmutated IGHV in comparison to mutated IGHV (P=0.08) and with CD38+ compared to CD38- (P=0.062) (Fig. S3).

**Figure 4.**
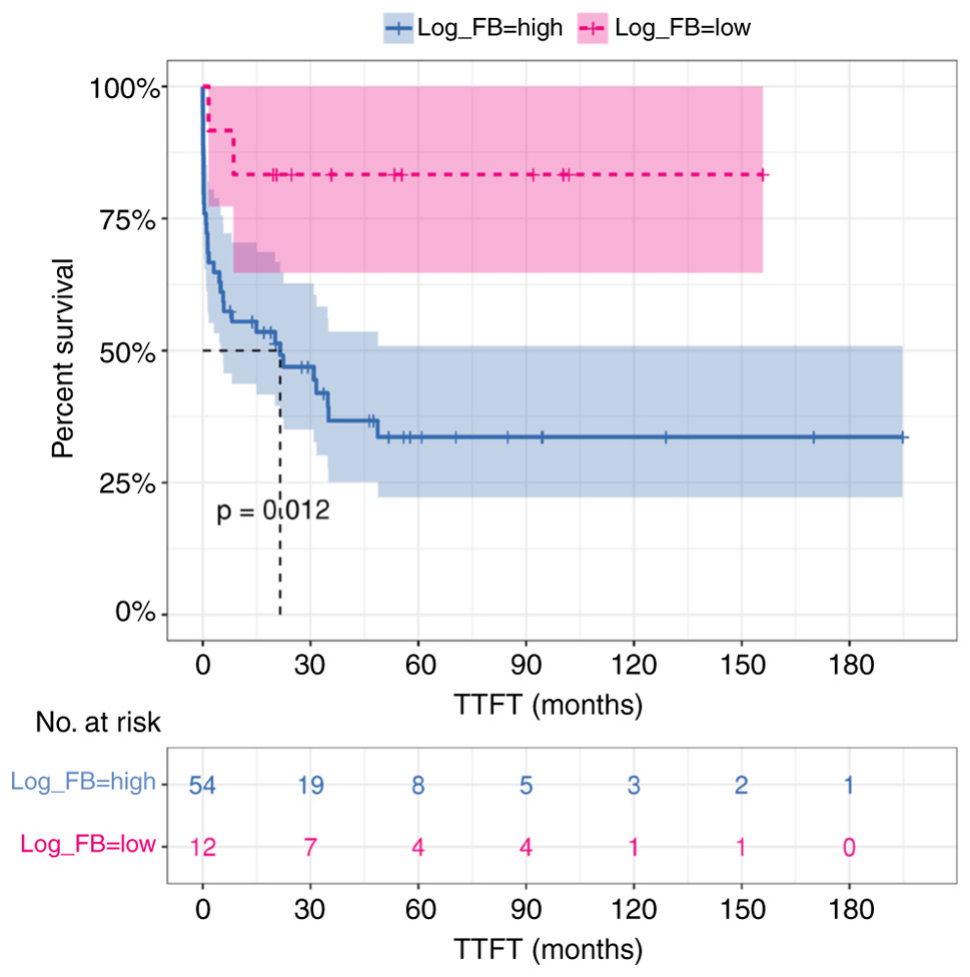
Intestinal log FB ratio and TTFT in patients with CLL (n=66). Subjects were categorized according to the values of log FB ratio in stool samples (cutpoint=−0.39, as calculated by maximally selected rank statistics). Kaplan-Meier survival curve is shown with 95% CI for log_FB=high (n=54) vs. log_FB=low (n=12). The P-value was calculated by Cox proportional hazard models (as implemented by the R survival package). TTFT, time to first treatment; FB, *Firmicutes/Bacteroidota*; CLL, chronic lymphocytic leukemia; HVs, healthy volunteers.

In the univariate model of Cox regression analysis, intestinal log F/B ratio <-0.39 (as calculated by maximally selected rank statistics) and corresponding to the 17th percentile, was a significant predictor of longer TTFT in CLL patients (HR 5.20, 95% CI 1.25–21.72, P=0.024) (Table II). However, the multivariate analysis, including established risk factors (Binet stage, IGHV mutation status, CD38 expression, del11q, isolated del13q), showed that fecal log F/B ratio had no impact on TTFT (HR 1.16, 95% CI 0.12–10.93, P=0.897). Kaplan-Meier estimate confirmed that intestinal microbiota dysbiosis, with log F/B ratio >-0.39, was associated with a significantly shorter TTFT (median 21.5 vs. NA, P=0.012) (Fig. 4).

## Discussion

A growing body of research has proved the significance of microbiome alterations and the potential role of specific microbial taxa in hematological malignancies (15–18,50,51). Analysis of oral and intestinal microbiota of newly diagnosed CLL patients in parallel with HVs allowed for the first time, to discover significant differences in bacterial composition in CLL patients. Loss of microbiome complexity in our CLL cohort was observed as a decreased abundance of *Bacteroidota* and, consequently, altered *Firmicutes/Bacteroidota* (F/B) ratio. Our findings are in line with previous reports of the reduced bacterial diversity and intestinal F/B ratio imbalance in other inflammatory conditions, including obesity, type 2 diabetes, inflammatory bowel disease, and cancers (52,53).

An enrichment of the *Proteobacteria* phylum was revealed, which is a marker of dysbiosis associated with intestinal inflammatory diseases, such as Crohn's disease and ulcerative colitis (54). While an increase in *Proteobacteria* may be linked to B cell differentiation, the underlying mechanism is still unclear (55). Lipopolysaccharide (LPS), which is a component of the outer membrane of *Proteobacteria*, through activation of Toll-like receptor 4 (TLR4), triggers downstream signaling pathways, including NF-κB activation and dysregulates BCR signaling that represents a stimuli factor driving CLL cells into proliferation (56). Moreover, LPS-induced activation of TLR4 promotes inflammation by stimulating the production and release of pro-inflammatory cytokines (such as TNF-α, IL-1β, and IL-6), thereby indirectly contributing to neoplasia through maintaining proinflammatory microenvironment (57,58). Thus, increased LPS levels from *Proteobacteria* may contribute to sustained immune activation and inflammation, potentially affecting the pathogenesis of CLL. Yuan *et al* (59) showed an increased relative abundance of *Proteobacteria* and a continuous evolutionary relationship of gut microbiota, from *Proteobacteria* phylum to *Escherichia-Shigella* genus, in untreated diffuse large B-cell lymphoma (DLBCL) patients. Moreover, *Proteobacteria* was more abundant in the intestinal microbiome of multiple myeloma (MM) patients as compared to healthy controls (60). Interestingly, this study also showed an enrichment of nitrogen-recycling bacteria from the *Proteobacteria* phylum, such as *Klebsiella*, in the microbial community structure of MM patients. These bacteria are involved in the hydrolysis of urea, which accumulates in excessive amounts in the blood and intestines in MM patients, and the synthesis of L-glutamine, which is taken up by MM cells, thereby may promote tumor progression.

SCFAs, which include acetate, propionate, and butyrate, are generated through the fermentation of non-digestible carbohydrates by anaerobic bacteria (phylum *Bacteroidota* for propionate and acetate; phylum *Firmicutes* for butyrate) in the colon (61). These biologically active microbial metabolites are key regulators of host physiology, including immune system balance via promoting both immune response and tolerance (62). SCFAs suppress nuclear factor κB and inhibit the production of proinflammatory cytokines such as IL-6 and TNF-α (63). Moreover, SCFAs upregulate anti-inflammatory IL-10 via activation of aryl hydrocarbon receptor (AhR)-dependent gene transcription in B cells and promotion of regulatory B cells differentiation (64). SCFAs promote the generation of Th1, Th17, and Treg through the inhibition of histone deacetylase (HDAC) activity (65). The HDAC mechanism is also involved in initiating Fas-mediated T cell apoptosis by butyrate. At lower concentrations, butyrate inhibits T cell proliferation, while at higher concentrations, it induces apoptosis of activated T cells. Consequently, the accumulation of T cells within the inflamed colonic mucosa is inhibited, which eliminates potential antigenic stimulation and results in inflammation (66,67). Dysregulation of the innate and adaptive immune system is a crucial feature in CLL patients. Immunosuppressive signatures include expansion of anti-inflammatory cells such as Treg and myeloid-derived suppressor cells, production of immunosuppressive soluble factors such as IL-10 and TGF-β, and functional exhaustion of CD8+ effector T cells through expression of inhibitory receptors (programmed death receptor-1 (PD-1), cytotoxic T lymphocyte antigen-4 (CTLA-4), lymphocyte activation gene 3 (LAG-3), CD244 and CD160) (68).

Notably, alterations in microbiota composition in CLL patients in our study were related mainly to *Lachnospiraceae, Ruminococcaceae, Prevotellaceae*, and *Veillonellaceae* families, which are among the main producers of SCFAs. In the recent study by Faitová *et al* (19), the abundances of SCFAs-producers belonging to *Lachnospiraceae* and *Ruminococcaceae* families in the gut microbiota of CLL patients were found to be significantly lower in comparison to healthy control. However, this observation was limited to a small group of CLL individuals. Since CLL itself is a heterogeneous disease with varying clinical course and progression rates, the disease stage may differentially impact the microbiota composition. In our study, oral and gut microbiota composition in 81 CLL patients at different stages of the disease was analyzed: 30 CLL patients (37%) were at Binet stage A, 23 CLL patients (29%) were at Binet stage B, and 25 CLL patients (31%) were at Binet stage C. For 3 CLL patients (3%), Binet stage data were not available. In contrast, the CLL cohort in Faitová's study (19) included 70% of CLL patients with Binet stage A, 20% of CLL patients with Binet stage B, and 10% of CLL patients with Binet stage C. In our CLL cohort, we found significant differences in oral and gut microbiome structure depending on the disease stage. In Faitová's study (19), the size of the CLL cohort was limited, and microbiome alterations between CLL patients at different disease stages were not analyzed. Additionally, our CLL cohort consisted of 47% of patients with mutated IGHV and 40% with unmutated IGHV, while in Faitová's study (19), 70% of patients were unmutated-CLL, and 30% were mutated-CLL. Therefore, the molecular prognostic features, which include IGHV mutation status, indicate that our CLL patients group is the diagnostic cohort, and the CLL patients group in Faitová's study (19) is the cohort requiring treatment initiation. As we excluded patients who had a course of antibiotics within 4 weeks before sampling, it is noteworthy that non-antibiotic drugs like non-steroidal anti-inflammatory drugs (69), calcium-channel blockers (70), and antipsychotics (71) can also affect the human microbiome. Moreover, dietary habits and geographical location may shape the diversity and composition of the microbiome (72,73). An important issue in microbiome analysis remains methodological differences such as variations in DNA extraction methods, library preparation, sequencing platforms, and pipelines (74,75).

In addition to modulation of tumor growth, the gut microbiota can affect the response to treatment. MM patients with minimal/measurable residual disease negative response after completion of upfront therapy showed a higher relative abundance of SCFAs-producers *Eubacterium hallii* and *Faecalibacterium prausnitzi* (76). Additionally, a decreased risk of MM relapse/progression after allogeneic hematopoietic cell transplantation was associated with the enrichment of *Eubacterium limosum* according to the study by Peled *et al* (77). Yoon *et al* (78) showed a positive correlation of *Escherichia, Klebsiella, Lactobacillus*, and *Weissella* genera abundance in the gut microbiome of DLBCL patients with indicators predicted to be associated with disease burden, such as Ann Arbor stage and international prognostic index, as well as a higher susceptibility to side effects of chemotherapy.

Nevertheless, this study suggests the multi-faceted role of microbiome in the pathology of CLL. For instance, SCFA-producing bacteria, whose abundance was significantly changed in our CLL cohort, have been previously reported to be implicated in the maintenance of the intestinal barrier integrity (18,79,80), regulation of macrophage balance in the intestine (81), and modulation of NK cell cytotoxicity activity (82). Moreover, by analyzing both oral and gut microbiota in CLL patients and observing a loss of complexity and remarkable differences in the relative abundances of specific bacterial phyla, a CLL-specific microbiome profile characterized by enrichment of *Proteobacteria*, depletion of *Bacteroidota*, and impaired F/B ratio was identified. We observed a higher value of log F/B ratio in CLL patients with more advanced disease stage and negative prognostic features (high CD38 expression and unmutated IGHV) and the association of an increased value of this parameter with a significantly shorter TTFT, which is in line with Faitova *et al* study (83) showing that lower diversity of the gut microbiome in CLL patients is associated with more aggressive and/or more progressive disease development. Although we aimed for our CLL cohort to be as homogenic as possible, not all alterations observed in the oral and intestinal microbiome were the same. These differences may be related to specific factors associated with each anatomical site. The oral cavity plays a key role in host defense against invading antigens and is directly exposed to external factors such as diet and oral hygiene practices (84,85). In contrast, the gut microbiome is more complex and diverse than the oral microbiome (86). Furthermore, CLL is characterized by immune dysregulation, which can lead to alterations in the local immune environment of both the oral cavity and the gut. However, this immune dysregulation may affect microbial communities differently in each site.

In conclusion, the structure of oral and gut microbiota of CLL patients exhibits specific alterations in comparison to healthy individuals and is associated with distinct prognostic features. Furthermore, our findings suggest that altered microbiota might be implicated in CLL pathogenesis through SCFA-related metabolic pathways, thus intestinal microflora modulation might provide a novel approach to improve the efficacy of CLL treatment.

## Supplementary Material

Supporting Data

Supporting Data

Supporting Data

Supporting Data

## Figures and Tables

**Table I.** Clinical characteristics of patients with chronic lymphocytic leukemia.

Characteristic	No. of patients
Age (median, range)	65 (33–85)
Sex, n (%)	
Female	32 (40%)
Male	49 (60%)
Binet stage, n (%)	
A	30 (37%)
B	23 (28%)
C	25 (31%)
Not available	3 (4%)
CD38 (cut-off 30%), n (%)	
Positive	35 (43%)
Negative	43 (53%)
Not available	3 (4%)
ZAP-70 (cut-off 20%), n (%)	
Positive	18 (22%)
Negative	60 (74%)
Not available	3 (4%)
IGHV mutation status, n (%)	
Mutated	38 (47%)
Unmutated	32 (40%)
Not available	11 (13%)
BCR immunoglobulin stereotypy, n (%)	
Stereotyped subsets (major and minor)	25 (47%)
High risk stereotyped subsets (#2, #5, #8b)	5 (7%)
Not available	11 (14%)
*TP53* mutation status, n (%)	
Mutated	5 (6%)
Wild-type	74 (92%)
Not available	2 (2%)
Cytogenetics, n (%)	
del11q	19 (24%)
del13q	48 (60%)
isolated del13q	30 (37%)
del17p	6 (7%)
tri12	9 (11%)
del6q	3 (4%)
Not available	1 (1%)

IGHV, immunoglobulin heavy chain gene; BCR, B cell receptor.

**Table II.** Univariate and multivariate analyses of time to first treatment in the cohort of 66 patients with chronic lymphocytic leukemia.

	Univariate		Multivariate	
		
Variable	HR (95% CI)	P-value	HR (95% CI)	P-value
Binet stage, A vs. B	11.60 (2.63–51.08)	0.001	13.82 (1.44–132.53)	0.023
Binet stage, A vs. C	27.89 (6.40–121.45)	<0.001	54.56 (5.22–568.32)	<0.001
CD38 expression, positive vs. negative^a^	3.33 (1.66–6.67)	<0.001	0.53 (0.16–1.80)	0.311
IGHV, unmutated vs. mutated	0.14 (0.06–0.034)	<0.001	0.52 (0.15–1.84)	0.307
Del11q, present vs. absent	4.49 (2.22–9.10)	<0.001	2.51 (0.99–6.38)	0.052
Isolated del13q, present vs. absent	0.39 (0.18–0.85)	0.019	0.48 (0.14–1.65)	0.243
Intestinal log FB^b^	5.20 (1.25–21.72)	0.024	1.16 (0.12–10.93)	0.897

a Cut-off 30%.

b Cut point at −0.39. FB, *Firmicutes/Bacteroidota*; IGHV, immunoglobulin heavy chain gene; CI, confidence intervals.

## Data Availability

The raw 16S rRNA sequencing data have been deposited in the European Nucleotide Archive at EMBL-EBI under accession number PRJEB67303 (www.ebi.ac.uk/ena/browser/view/PRJEB67303). All other data generated in the present study may be requested from the corresponding author.
