# Supplementary material for: Increased abundance of Firmicutes and depletion of Bacteroidota predicts poor outcome in chronic lymphocytic leukemia
Source: Oncol Lett. 2024 Sep 17;28(5):552. doi: 10.3892/ol.2024.14685 (PMC11425030; doi:10.3892/ol.2024.14685)

Figure S1. Flow cytometric analysis of CD38 and ZAP-70 expression on CLL cells. Peripheral blood cells of patients with CLL were analyzed after staining with anti-CD19 FITC, anti-CD5 PE-Cy5, anti-CD38 PE and anti-ZAP-70 PE antibodies. (A) Gating strategy used to select lymphocytes in the left plot and CLL cells (CD19 + CD5+) upon gating on lymphocytes in the right plot. (B) Plots showing a representative CD38-positive sample in the right plot and FMO control in the left plot. (C) Plots showing a representative ZAP-70-positive sample in the right plot and FMO control in the left plot. The numbers show the proportion of cells positive for indicated markers. CLL, chronic lymphocytic leukemia; HVs, healthy volunteers; FMO, fluorescence minus one.

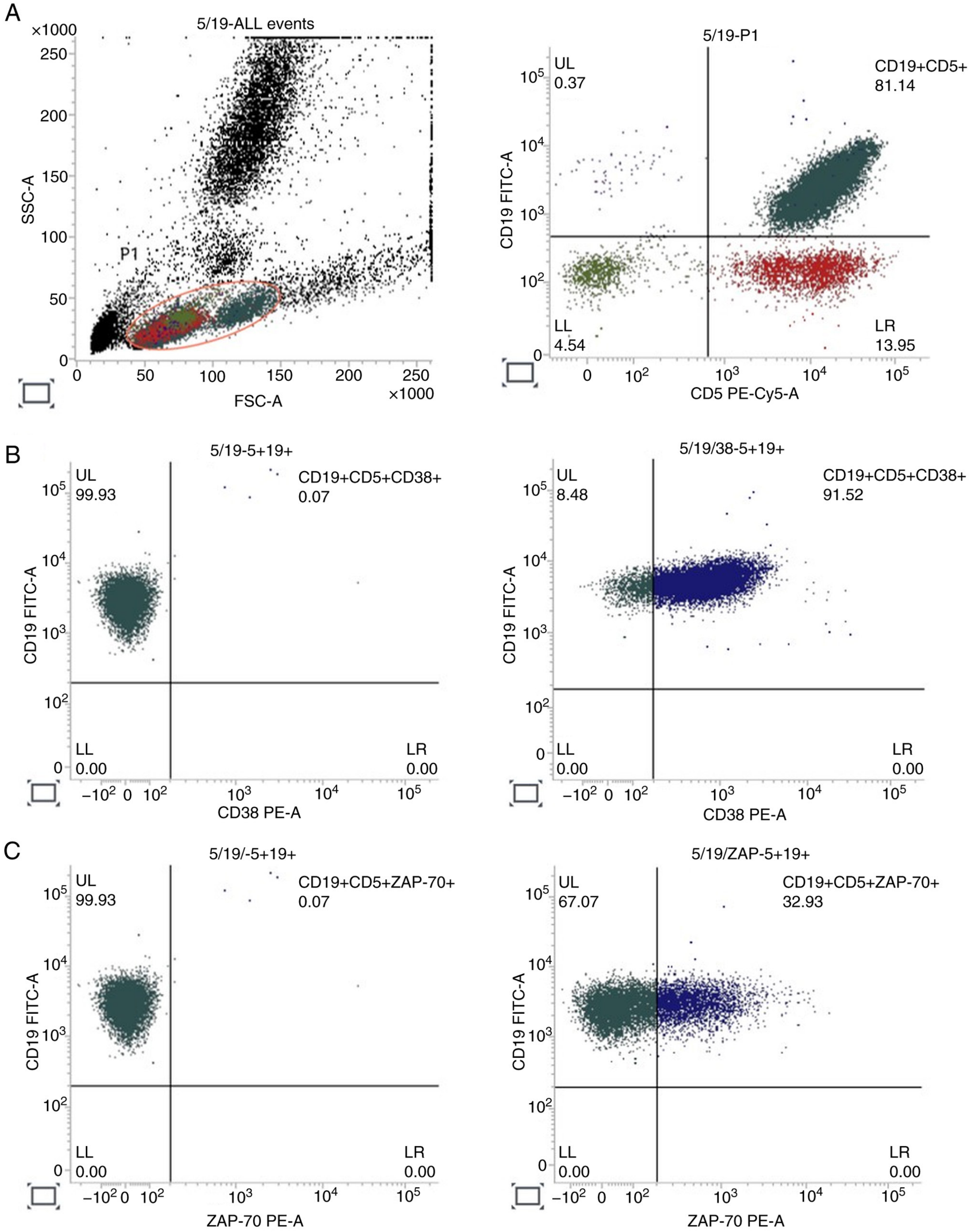

Figure S2. Distribution of the most dominant bacterial phyla in oral and fecal samples from patients with CLL and HVs. Distribution of the dominant bacterial taxa at the phylum level in oral samples from (A) patients with CLL and (B) HVs, and in gut samples from (C) patients with CLL and (D) HVs. CLL, chronic lymphocytic leukemia; HVs, healthy volunteers.

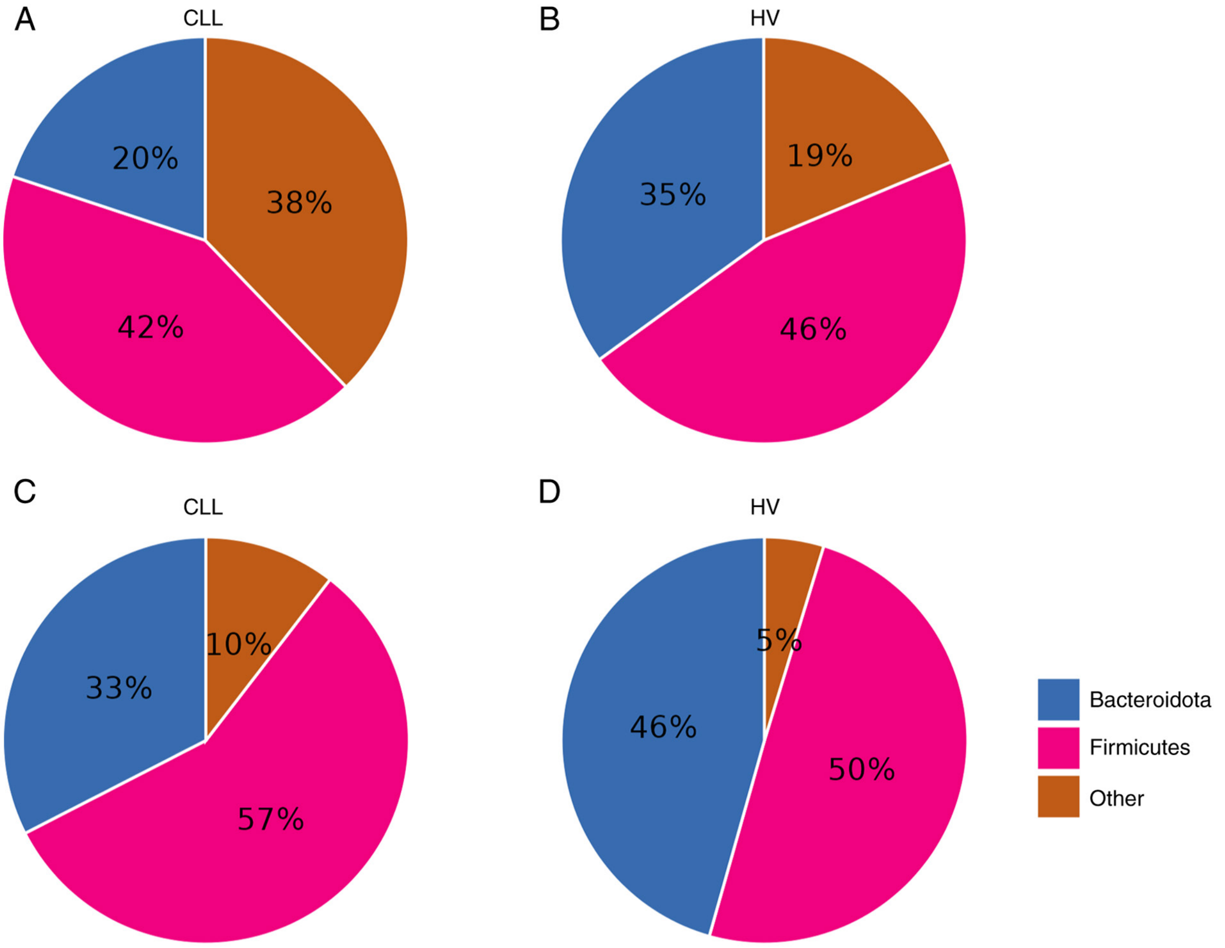

Figure S3. Log FB ratio in fecal samples from patients with CLL with distinct prognostic factors. (A) Binet stage: Binet stage A (n=27), Binet stage B (n=22), Binet stage C (n=23), Binet stage B vs. Binet stage C (P=0.581). (B) CD38 expression: Positive (n=31) vs. negative (n=41). (C) ZAP-70 expression: Positive (n=15) vs. negative (n=57). (D) IGHV mutation status: Unmutated (n=28) vs. mutated (n=36). (E) TP53 mutation status: Mutated (n=5) vs. wild-type (n=65). (F) BCR immunoglobulin stereotypy: High risk stereotyped subsets (#2, #5, #8b) present (n=6) vs. absent (n=18). Cytogenetic aberrations: (G) del11q present (n=16) vs. absent (n=58); (H) isolated del13q present (n=28) vs. absent (n=46); (I) tri12 present (n=7) vs. absent (n=67). The P-values were calculated by a two-sided Wilcoxon rank-sum test without multiple comparison adjustments. CLL, chronic lymphocytic leukemia; HVs, healthy volunteers; FB, *Firmicutes*/*Bacteroidota*.

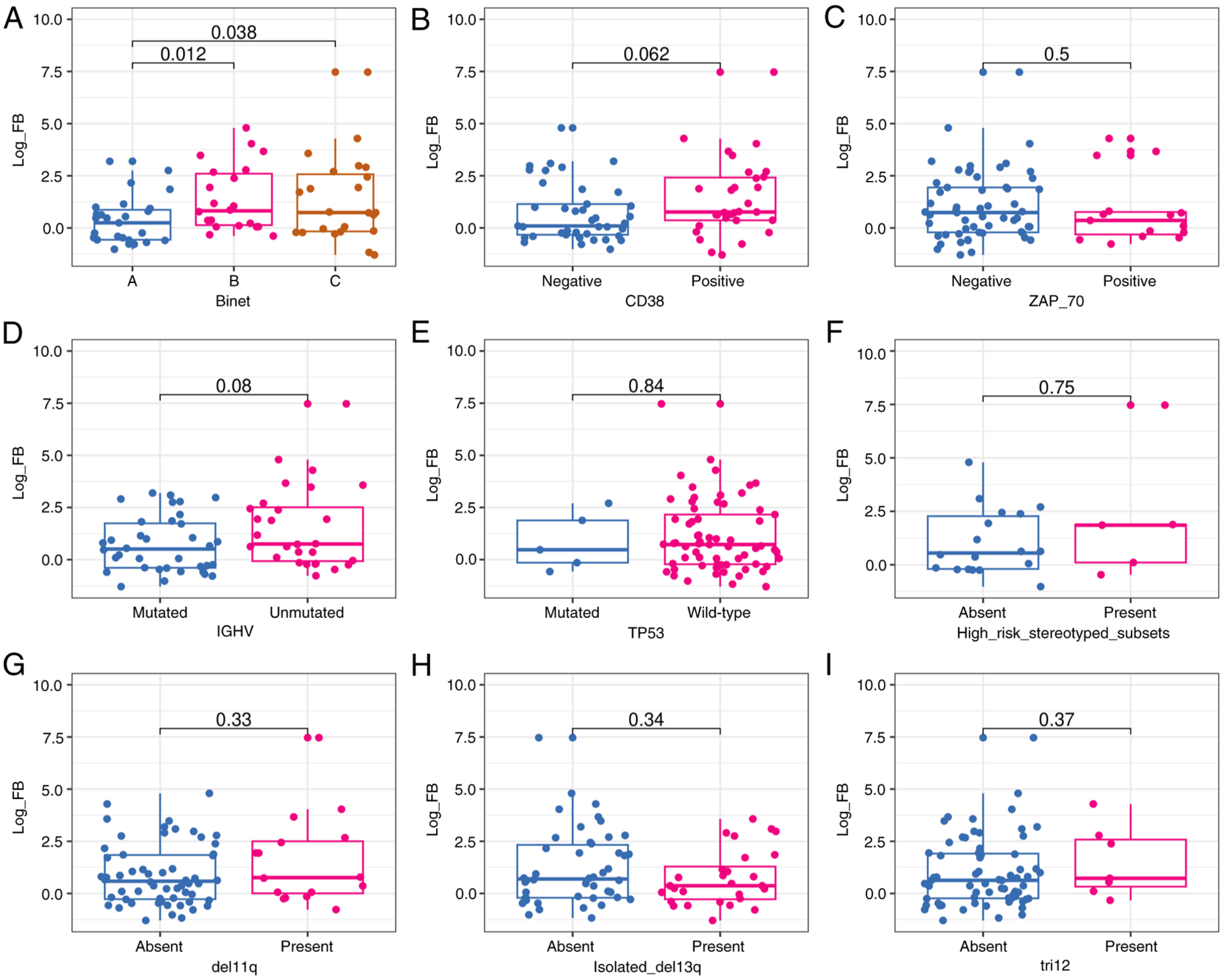

Supplement: Supporting Data [file Supplementary_Data1.pdf]
