# Supplementary material for: Increased abundance of Firmicutes and depletion of Bacteroidota predicts poor outcome in chronic lymphocytic leukemia
Source: Oncol Lett. 2024 Sep 17;28(5):552. doi: 10.3892/ol.2024.14685 (PMC11425030; doi:10.3892/ol.2024.14685)
Supplement: Supporting Data [file Supplementary_Data2.pdf]

Table SI. Relative abundance composition of the oral microbiota in patients with CLL and HVs.

	CLL		HVs		
Phylum	Median relative abundance (%)	Range	Median relative abundance (%)	Range	P-value
Actinobacteriota	6.85	0.08-93.34	5.36	2.00-11.05	0.14
Bacteroidota	16.35	0.00-83.79	37.05	1.31-56.78	0.0015
Firmicutes	40.53	1.18-93.41	46.39	25.43-84.07	0.45
Fusobacteriota	6.50	0.00-29.08	5.11	0.00-25.74	0.63
Proteobacteria	8.07	0.14-94.19	2.39	0. 11-20.88	0.022
Other[a]	0.66	0.00-1.41	0.31	0.00-0.64	

[a]Phyla with a relative abundance <0.1% in each sample were merged into ‘Other’. CLL, chronic lymphocytic leukemia; HV, healthy volunteers.

Table SII. Relative abundance composition of the gut microbiota in patients with CLL and HVs.

	CLL		HVs		
Phylum	Median relative abundance (%)	Range	Median relative abundance (%)	Range	P-value
Actinobacteriota	0.75	0.01-26.37	0.92	0.22-3.83	0.85
Bacteroidota	31.41	0.04-75.88	43.04	18.06-84.27	0.026
Firmicutes	58.01	20.79-91.98	53.89	14.56-79.07	0.18
Proteobacteria	2.73	0.10-39.07	1.64	0.47-6.54	0.045
Other[a]	1.09	0.00-2.25	0.96	0.13-3.85	

[a]Phyla with a relative abundance <0.1% in each sample were merged into ‘Other’.
